# Effect of Nano- and Microparticulate Fermented Fish Protein Hydrolysate Diets on Growth, Digestive Function, Intestinal Development, and Gene Expression in Common Carp (*Cyprinus carpio*) Larvae

**DOI:** 10.3390/biology15191680

**Published:** 2026-09-22

**Authors:** Zohaib Noor, Faisal Ahmad Lodhi, Waqar Younas, Fahim Ullah Khan, Zaigham Hassan, Muhammad Rizwan, Saif Ullah, Fida Ullah Khan, Fathul Bari, Md. Fakhrul Islam

**Affiliations:** 1 School of Marine Biology and Fisheries, Hainan University, Haikou 570228, China; 2 School of Life and Health Sciences, Hainan University, Haikou 570228, China; 3 Department of Zoology, Quaid-i-Azam University, Islamabad 45320, Pakistan; faisalahmadlodhi2000@gmail.com (F.A.L.);; 4 Key Laboratory of Exploration and Utilization of Aquatic Genetic Resources, Ministry of Education, College of Fisheries and Life Science, Shanghai Ocean University, Shanghai 201306, China; 5 State Key Laboratory of Breeding Biotechnology and Sustainable Aquaculture, Institute of Hydrobiology, Chinese Academy of Sciences, Wuhan 430072, China; fakhrul@jstu.ac.bd; 6 University of Chinese Academy of Sciences, Beijing 100049, China; 7 Department of Zoology, University of Peshawar, Peshawar 25000, Pakistan; 8 College of Fisheries, Huazhong Agriculture University, Wuhan 430070, China; 9 Department of Zoology, Islamia College Peshawar, Peshawar 25000, Pakistan; 10 Department of Zoology, University of Chitral, Chitral 17200, Pakistan; 11 Department of Fisheries, Jamalpur Science and Technology University, Jamalpur 2012, Bangladesh

**Keywords:** larval feed, nanoparticulate feed, fish protein hydrolysate, fermented feed, digestive enzymes, gene expression

## Abstract

The early larval stage is a major bottleneck in freshwater aquaculture because conventional live feeds are not always available for optimum nutrient availability and digestibility to satisfy the nutritional requirements of developing larvae. This study evaluated the effects of micro- and nano-particulate fermented and fish protein hydrolysate-based diets on growth, digestive enzyme activity, intestinal morphology, and immune-related gene expression in common carp (*Cyprinus carpio*) larvae. Four experimental diets, namely fish hydrolysate-based micro-feed (FHMF), fermented micro-feed (FMF), fish hydrolysate-based nano-feed (FHNF), and fermented nano-feed (FNF), were compared with a basal diet. Feed characterization confirmed the successful production of micro- (5–15 µm) and nano-sized (90–105 nm) particles with distinct physicochemical properties. Three-day-old hatchlings were reared for 45 days, and growth performance, survival, digestive enzyme activities, intestinal histomorphology, and gene expression were analyzed. Diet type significantly influenced all measured parameters (*p* < 0.001). Among the treatments, FNF consistently produced the highest growth, survival, digestive enzyme activities, villus development, and muscular layer thickness. Growth-related genes (GH, IGF-1, and ghrelin) and immune-related genes (lysozyme, TNF, IL-1β, IL-10, and TGF-β) also followed an upregulated trend. These findings demonstrate that nanoparticulate fermented feed is a promising nutritional strategy for enhancing larval growth, digestion, immune competence, and production efficiency in common carp aquaculture.

## Simple Summary

The larval period is an important and very sensitive stage in the culture of freshwater fish, where the young digestive system and low enzymatic activity in the larvae may limit the efficient use of conventional formulated feeds, which can lead to poorer growth performance and survival. In this study, we developed micro- and nano-sized diets using fermented ingredients and fish protein hydrolysates and evaluated their effects on common carp larvae stages. Among the tested diets, the nano-particulate fermented feed produced the best results by improving growth, survival, digestive enzyme activity, intestinal development, and the expression of genes associated with growth and immunity. The smaller particle size likely enhanced nutrient availability and absorption, allowing larvae to utilize nutrients more efficiently during early development. These findings suggest that nano-particulate fermented diets can provide a practical and sustainable feeding strategy to improve larval quality, increase survival, and enhance production efficiency in common carp aquaculture. This approach may also benefit the culture of other freshwater fish species during early development.

## 1. Introduction

Common carp, an omnivorous fish, primarily feeds on sediment and organic debris [1]. In its early stages, it relies on Diptera, such as Lepidoptera and Hemiptera, and the main plant-derived food items belong to the families Poaceae, Araceae, and Hydrocharitaceae, serving as the predominant food source [2]. The long-term development of aquaculture requires access to high-quality seeds and feed tailored to the species’ development phases [3]. The early-stage feed serves as a baseline for fish development, as it is influenced by the ease of obtaining and breaking down nutrients [4].

In Pakistan, aquaculture is in its early stages, focusing mainly on carp, especially major carp. Typically, post-larvae are raised in earthen nursery ponds on live feed obtained by conventional methods, i.e., animal manuring [5]. However, challenges such as low survival rates, delayed growth, and low-quality seeds hinder the sustainable culture of these species [6]. Live feeds have several inherent limitations, including seasonal and spatial fluctuations in zooplankton and phytoplankton composition, inconsistent and potentially imbalanced nutritional profiles [7], and the occurrence of large-sized or predatory zooplankton that may be unsuitable or detrimental to larval fish [8]. Additionally, the fixed nutrient profile of live feed makes it difficult to adjust its nutrient content to meet the specific dietary needs of fish [9]. Despite these limitations, live feed remains widely used in the early rearing of major carps in Asian aquaculture; therefore, it is important to develop a prepared nano-diet for the early-stage fry that is both environmentally and physiologically acceptable for fish welfare.

The nutritional needs of fish change quantitatively and qualitatively during their development, necessitating a balanced diet that includes the right proportions of protein, fats, and carbohydrates [10]. Early feeding practices significantly influence the growth and maturity of fish [11]. However, little work has been done on the prepared diet of larvae for freshwater fish [12]. Developing micro- and nano-diets for fish larvae is not limited to finding the right nutrient combination. Still, it should include considerations about the ontogenetic development of the digestive system, direct or indirect activation of digestive enzymes, and digestibility of the different components of the diet [13]. Although some freshwater fish larvae can be fed formulated diets as early as mouth opening, live feed is substituted with compound diets only after some weeks of life in marine fish in the hatchery [12]. However, the early introduction of formulated diets as sole feed sources has met with limited success, even in freshwater species. Since the development of formulated compound larval diets around 1980 [14], research has increasingly focused on the nutritional requirements of freshwater fish larvae. As different early-stage feeds have been prepared and tested in the past and are ongoing, but no significant outcomes have been formulated that solidify the foundations for the best larval diet, we aimed to evaluate the best Larval diet by utilizing nanotechnology for preparing nano- and micro-diets with known crucial ingredients that could improve the health status and survivability of *C. carpio*.

The digestibility and nutrient availability of the ingredients can be enhanced by hydrolysis and fermentation [15]. Fermented feeds play a crucial role in enhancing the nutritional profile of fish diets by increasing the availability of amino acids and reducing antinutritional factors [16]. The substantial carbon and oxygen content in larval feeds serves as a vital energy source, essential for meeting the high metabolic demands of growing larvae [17]. Additionally, hydrolysate-based feeds are rich in bioactive peptides, which have been shown to improve developmental performance and boost the immunological responses of fish larvae [18].

These advancements aim to provide a sustainable and efficient alternative to live feed, ensuring the long-term viability of aquaculture. It is asserted that fermentation and hydrolysis significantly enhance the digestibility and nutritive value of larval feed, based on their use in previous studies but regarding the Larval diet, and especially *C. carpio*, limited data are available. Therefore, the current study was designed to briefly study the detailed outcomes of the nano and micro diets and their influence on the physiological indices, gene expression, and growth rate of Larval stages.

## 2. Materials and Methods

### 2.1. Experimental Study Area

The experiment was conducted outdoors at the Fisheries and Aquaculture Research Station (FARS), Department of Zoology, Faculty of Biological Sciences, Quaid-i-Azam University, Islamabad, Pakistan.

### 2.2. Experimental Larvae

The Rawal Fish Seed Hatchery, Islamabad, provided 5000 hatchlings (post-larvae) of *C. carpio*, which were three days old. These hatchlings were delivered to the research facility in oxygenated polythene bags within 30 min and then stocked in the required experimental fiberglass tanks.

### 2.3. Fish Meal Hydrolysate Preparation

Fish protein hydrolysate formation was carried out by thawing the frozen fish (meat). To deactivate the meat’s natural enzymes, 200 g of the meat was manually ground, then twice as much water was added and heated to 85 °C for 20 min. The boiled meat was allowed to cool to room temperature before enzymatic hydrolysis. A 0.01% solution of papain was then added, and the mixture was incubated at 37 °C for 120 min at a pH of 7.0. To hydrolyze the protein content. The mixture was heated to 85 °C for 15 min to complete the hydrolysis. The hydrolyzed fish protein was then filtered. The liquid that made it through the filter was then centrifuged for 20 min at 6000 rpm. For future use, the soluble portion was freeze-dried and kept at 4 °C.

#### Determination of Degree of Hydrolysis

The degree of hydrolysis (DH) of the fish protein hydrolysate was determined using the o-phthaldialdehyde (OPA) method, based on the reaction of OPA with primary amino groups released during proteolysis. The OPA reagent was prepared by dissolving 7.62 g of sodium tetraborate decahydrate and 200 mg of sodium dodecyl sulfate (SDS) in approximately 150 mL of distilled water. Separately, 160 mg of OPA was dissolved in 4 mL of 96% ethanol and added to the borate/SDS solution, followed by 176 mg of dithiothreitol (DTT); the final volume was adjusted to 200 mL with distilled water, and the reagent was stored in an amber bottle at 4 °C until use.

A standard curve was constructed using L-serine solutions (0, 0.5, 1.0, and 1.5 mM) prepared in distilled water. Freeze-dried hydrolysate samples were dissolved in distilled water at an appropriate dilution (1–5 mg protein/mL) to give an absorbance within the linear range of the assay. For each sample and standard, 200–400 μL was mixed with 3 mL of OPA reagent, vortexed, and incubated for exactly 2 min at room temperature in the dark, after which absorbance was measured at 340 nm against a reagent blank using a UV–Vis spectrophotometer. All samples and standards were assayed in triplicate.

The concentration of free amino groups (serine-NH2 equivalents) in each sample was calculated from the serine standard curve. The degree of hydrolysis was then calculated according to the formula::

DH (%) = (h/htot) × 100

where h is the number of hydrolyzed peptide bonds (meqv/g protein), calculated from the serine-NH2 equivalent using h = (Serine-NH2 − β)/α, with α = 1.00 and β = 0.40 as constants appropriate for fish protein substratesand htot is the total number of peptide bonds in the substrate protein, taken as 8.6 meqv/g protein for fish muscle protein. The degree of hydrolysis was 7.2 ± 1.8%. Hydrolysis conditions: pH 7.0, 37 °C, 120 min, and Enzyme Papain (0.01% *w*/*v*).

### 2.4. Formulation and Preparation of Micro- and Nano-Particulate Feed

The food was formulated and prepared, containing 50% crude protein [17]. In accordance with the ratio specified in Tables S1 and S2, the dried components were first individually ground finely in an electric grinder (Model HC-500, Huanghua Faithful Instrument Co., Ltd., Cangzhou, China). To create pellets, the dough was made by adding water and then grinding it in a meat grinder. The pellets were dried at 50 °C in an electric oven and then stored at 4 °C. A ball mill grinder was used to crush and turn the previously used pellets into micro- and nanoparticles. Briefly, it had a barrel that held nine large and twenty-four small balls, and it was filled with 200 g of prepared feed that had been finely ground to turn it into nanoparticles. Two different kinds of 50% CP experimental diets were created: the first diet (HF) used protein hydrolysate as its main protein source, while the second diet (FF) used probiotic *Saccharomyces cerevisiae* NDS (LZB 105) to ferment a diet based on fish meal.

#### 2.4.1. Hydrolysate Feed Formulation

The Hydrolysate was formulated as shown in Table S1, in which fishmeal was replaced with fishmeal hydrolysate as the protein source; the process was the same as for particulate formulation.

#### 2.4.2. Fermented Feed Formulation

*Saccharomyces cerevisiae* NDS (LZB 105) was obtained from the Quaid-i-Azam University Laboratory for Microbial Food Safety and Nutrition. The 50% CP fish meal-based larval diet was formulated, and all the feed ingredients shown in Table S2 were mixed at a fixed ratio and autoclaved, as were the flask and tap water. The autoclaved feed was then fermented with *Saccharomyces cerevisiae*. The feed substrate was first weighed and adjusted to the desired initial moisture content, maintained within the optimal range of 40–60% (*w*/*w*) for SSF. The required moisture level was achieved by adding a calculated volume of sterile distilled water, followed by thorough homogenization to ensure uniform moisture distribution throughout the substrate. To eliminate indigenous microbial contaminants, the moistened substrate was sterilized by autoclaving at 121 °C for 15–20 min. After sterilization, the substrate was allowed to cool to room temperature (approximately 30 °C) under aseptic conditions. The cooled substrate was then inoculated with a freshly prepared *S. cerevisiae* suspension at an inoculation rate of 5% (*v*/*w*; mL of inoculum per 100 g of dry substrate). The mixture was thoroughly mixed to ensure even distribution of yeast cells within the substrate matrix. The inoculated material was transferred into sterile, breathable fermentation flasks to facilitate aerobic conditions, which are favorable for yeast growth and metabolic activity. Fermentation was conducted in an incubator maintained at 30 °C for 72 h. At the end of the fermentation period, the process was terminated by oven-drying the fermented substrate at 50–60 °C until a constant weight was achieved. This step effectively deactivated the yeast cells and prevented further fermentation. The dried fermented product was subsequently stored and used for fish feeding trials. The Hydrolysate was formulated with fishmeal replaced by fishmeal hydrolysate as the protein source; the entire process was the same as that used for the particulate formulation (Table S1).

### 2.5. Feed Characterization

The feed was examined by X-ray diffraction (XRD) at the National Centre for Physics (NCP) in Islamabad using a wavelength range of 0.7 to 2.3 A. This analysis focused on determining the size and structure of the particles as well as the dimensions, form, and surface characteristics of the feed particles using a scanning electron microscope (SEM) (JSM, 6490-LA, JEOL Ltd., Tokyo, Japan) at a magnification of 20,000×. Additionally, a scanning electron microscope (JSM-6490-LA, JEOL Ltd., Tokyo, Japan) at 20,000× magnification was employed to examine the morphology, dimensions, and surface characteristics of the feed particles. Furthermore, Fourier-Transform Infrared Spectroscopy (FTIR) was conducted to identify the functional groups present in the experimental diet, while Energy-Dispersive X-ray Spectroscopy (EDX) was used to determine the elemental composition of the feed particles.

### 2.6. Proximate Composition of the Experimental Diet and Larvae Fed

Proximate composition was conducted using an accredited laboratory facility at Poultry Research Institute (PRI), and standard protocols [19] were adopted. The micro Kjeldahl method was used for the estimation of crude protein, while the Soxhlet apparatus was used for the determination of crude fat content. Ash was measured by incineration in a muffle furnace for 6 h at 550 °C, while dry matter was determined by drying the sample until a constant weight was achieved.

### 2.7. Experimental Design

Experimental design and setup were used as reported earlier by [20]. Small circular experimental tanks (troughs) with a capacity of 8.0 L were used for a 45-day feeding study in five replicates. They used a common control group. About 300 post-larvae 3 Days After Hatching (3DAH) per tank were randomly assigned to 25 small circular tanks for the experiment. As previously reported, the stocking density of 80 larvae per liter was determined using the methodology described by [21]. The tanks were randomly assigned to four groups: the Control group was raised on a 50% CP basal diet. In contrast, the T-1 group was reared on fish hydrolysate-based microfeed (FHMF), the T-2 group was reared on fermented microfeed (FMF), the T-3 group was reared on fish hydrolysate-based nanofeed (FHNF), and the T-4 group was reared on fermented nanofeed (FNF), respectively. The post-larvae were shifted to the glass aquaria after two weeks. For 45 days, each group was fed a particular diet. The larvae were initially fed frequently. However, during the feeding experiment, the frequency and rate of feeding were adjusted according to the body weight and growth of the fry. According to [20], the feed given during the feeding experiment was adjusted to the size of the larvae/DAH. By supplying a consistent supply of well-aerated water, water quality parameters were maintained throughout the experiment. The temperature of the water was found to be between 27.5 ± 0.5 °C and 28.0 ± 0.5 °C, pH of 7.5 to 8.5, dissolved oxygen levels above 5.0 mg/L, Nitrate was 0.32 ± 0.0128 mg L^−1^, nitrite 0.032 ± 0.00286 mg L^−1^, and 0.065 ± 0.00254 mg L^−1^. For the first 10 days, post-larvae were fed continuously at a rate of 75% of their body weight. After that, they were fed three to four times a day at a rate of 50% of their body weight for the next few days.

### 2.8. Sampling

Larvae and fry were sampled from each tank at 3, 10, 17, 24, 31, 38, and 45 days after hatching, depending on their size. This was carried out using a small bucket (1 L) with a hand net (40 μm mesh size) while partially opening the tank drain. Before feeding, sampling was carried out at 8:00 AM. Dead fry were removed every day, and the number of fry sampled was noted to calculate survival rates at the conclusion of the feeding trial. The number and wet weight of larvae/fry per tank were used for analyzing intestinal enzymes and gene expression. The procedures of [20,22] were followed in the collection of samples for histological and digestive enzyme analysis. Whole fry was pooled up to 10 DAH (9 samples per group). The fry was then dissected on an ice pad after being anesthetized with buffered MS-222 (75 mg/L). The abdominal section was taken up to 25 DAH, and the gastrointestinal (GI) tract was dissected and pooled at 35 DAH. For further analysis, the samples were immediately frozen in liquid nitrogen and kept at −20 °C. For gene expression analysis, a similar sample collection procedure was adapted, and samples were stored in RNA later™ (Thermo Scientific CAT#AM7020, Waltham, MA, USA) at 4 °C until further analysis.

### 2.9. Growth Performance and Survival

The growth performance and the survival rate of the larvae were analyzed using previously adapted procedures and formulas [23,24].

### 2.10. Intestinal Enzyme Assays

During the feeding trial, fries were fasted for 24 h before sampling to minimize digestive variability. Three fries were randomly collected from each tank (totaling 45 fries per treatment group) and anesthetized using buffered MS-222 (75 mg/L) before being dissected on an ice-cold surface. Approximately 0.5 g of intestinal tissue was excised and homogenized in 5 mL of ice-cold phosphate buffer (PBS; 0.1 M, pH 7.5) using an electric homogenizer (VWR POWER 200, VWR International, Radnor, PA, USA). The homogenate was then centrifuged at 10,000 rpm for 10 min at 4 °C (Eppendorf Centrifuge 5417R, Eppendorf SE, Hamburg, Germany), and the resulting supernatant containing crude enzyme extract was collected and stored at 4 °C for enzymatic analysis. The activities of intestinal enzymes, protease, lipase, and cellulase were assessed following standard protocols as outlined by [23].

### 2.11. Histological Study

For histological investigation, tissues of the experimental groups (Control, T1, T2, T3, and T4) were well-preserved, and slides were made by adopting the procedure previously described [25]. In brief, the dissected tissue was washed in phosphate-buffered saline (pH 7.2) to eliminate the blood and mucus. The tissue was then embedded in 10% formalin for a minimum of 24 h. The rest of the protocols were followed based on the previously adapted protocols [26].

### 2.12. Gene Expression (RNA Extraction and cDNA Synthesis)

For the transcriptomic expression of growth- and immune-related genes. The complete procedure of total RNA extraction (Roche, Basel, Switzerland) and cDNA synthesis was carried out in accordance with our previous work [25]. To check the purity of extracted RNA, NanoDrop-ND1000 (Tech. Inc., Wilmington, DE, USA) was used. Later, the isolated RNA was converted to cDNA using a cDNA synthesis kit (RevertAid First Strand cDNA synthesis kit) according to the manufacturer’s instructions (Thermo Scientific, Waltham, MA, USA).

Primers were designed (Table S3) with Oligo Primer Analysis Software version 1.1.2 and manufactured by Humanizing Genomics Macrogen. The nucleotide sequence of the respective genes of *Cyprinus carpio* was obtained from NCBI GeneBank (http://www.ncbi.nlm.nih.gov). A simple PCR followed by gel electrophoresis of each sample in duplicate was used to test the quality of the cDNA and its compatibility with primers. Following that, qPCR was carried out in accordance with the protocol described by [26]. The accuracy of the PCR reaction for each gene was determined by plotting standard curves with serial dilutions of a control sample’s cDNA (1/10, 1/100, and 1/1000) qRT-PCR protocol: denaturation initiates at 94 ◦C for 1 min, at 95 °C 40 cycles of amplification for 15 s, then 57 °C for 30 s, followed by 72 ◦C for 20 s, last for 1 min, and then signal collection for 15 s at 85 °C. After each reaction, dissociation curve analysis was conducted to confirm the specificity of the PCR products. The mRNA quantities for each gene were adjusted to *Cyprinus carpio* β-actin expression. The standard ΔΔCT method was used to determine the relative variations in gene expression [27].

### 2.13. Statistical Analysis

All experimental data were organized in MS Excel and analyzed using IBM SPSS (version 25; IBM Corp., Chicago, IL, USA). Initially, the data normality and homogeneity of variances were assessed using Shapiro–Wilk and Bartlett’s tests, respectively. All results are represented as mean ± SEM. Significant differences among experimentally treated groups were assessed using Two-way ANOVA followed by the LSD test using RStudio and SPSS Statistics version 21. For the graphical representation of the data, RStudio and GraphPad Prism 10 were used. For Histological examination, ImageJ (1.54r) was used. Pearson correlation analysis was conducted using RStudio statistical software Desktop Pro version 2025.09.2+418 (Posit Software, PBC, Boston, MA, USA, 2025). A significance level of 0.05 was established as statistically significant.

## 3. Results

### 3.1. Characterization of Micro- and Nano-Particulate Feed

#### 3.1.1. XRD Analysis of Experimental Diets

The X-ray diffraction (XRD) analysis showed different diffraction profiles for the four experimental diets (Figure 1A–D), suggesting that their physicochemical and structural properties differ. Table S4 summarizes the diffraction peaks and the corresponding 2θ for the principal peaks that have been identified for each of the dietary formulations. Most importantly, the four diets had different numbers, locations, sharpness, and sizes of their diffraction peaks. While the diffraction peaks of the microfeed formulations (FHMF and FMF) were well defined and relatively sharp, the nanofeed formulations (NF and FNF) exhibited fewer and less well-defined, but comparatively sharper diffraction peaks. These changes indicate there was a change in structural organization and degree of ordering of the molecules in the feed matrix caused by the processing treatments, such as hydrolysis, fermentation, and nano-processing. The less focused and broader diffraction features found in the nanofeed formulations could also suggest that processing has led to a decrease in the crystallinity of the structure and an increase in the disruption of the original feed matrix.

#### 3.1.2. Scanning Electron Microscopy (SEM) Analysis of Experimental Diets

The SEM shows clearly different particle size characteristics and surface morphology in the nano- and micro-particulate larval feeds (Figure 2A and Figure 2B, respectively) through scanning electron microscopy (SEM). The particle size distribution of the nano-particulate feed was relatively uniform, with the particle diameter of the feed in the range of 90.00 to 105.00 nm, while the micro-particulate feed was relatively coarse in particle size, from 5.00 to 15.00 µm. Although the difference in particle size is clear, both formulations displayed mostly smooth and relatively continuous surface morphologies, without any prominent cracks, pores, or irregular surfaces at the magnification used for the analysis. The small size of the nano-particulate size suggests that there was a significant reduction in particle size compared to the micro-particulate size, such that the feed matrix has a higher specific surface area and is more accessible to the digestive enzymes. These morphological features could be advantageous for improved hydration, dismemberment, and digestion in larva digestive tract, which should be evaluated by complementary digestibility and feeding-performance tests.

#### 3.1.3. Energy-Dispersive X-Ray (EDX) Analysis

Energy-dispersive X-ray spectroscopy (EDX) was used to characterize the elemental composition of the experimental feeds for the nano- and micro-particulate formulations (Figure 3A,B). Elemental spectra showed that there are distinct differences between the elemental profiles of the two feed formulations, which suggests that they differ in their elemental composition. The differences might be due to the different ingredients and processing procedures used in preparing the feeds, such as fermentation and hydrolysis. The processing-induced changes could affect the distribution or relative abundance of elements detected in the feed matrix and thus affect the physicochemical properties, nutrient availability, and functional performance of the particulate feed. EDX, however, does not give direct information on specific nutrient compounds, and therefore, the elemental differences observed should be understood as evidence of compositional differences, but not direct information on the change in nutrient availability.

#### 3.1.4. Fourier-Transform Infrared Spectroscopy (FTIR) Analysis

The FTIR spectra of experimental feeds are shown in Figure 4A–D, showing typical absorption bands that signify the existence of significant biochemical constituents and structural alterations among diets. The presence of proteins and hydrogen-bonded compounds was evidenced by a general band at 3200–3400 cm^−1^, whereas OH and NH stretching were at 2920–2850 cm^−1^, which is associated with the aliphatic CH stretching. There was a pronounced absorption at 17,001,650^−1^ (amide I) and FNF and FHNF around 1550 cm^−1^ (amide II), which had much stronger absorption in FNF and FHNF, indicating that the materials were proteinaceous, indicating protein hydrolysis and molecular interactions. Peaks between 1400 and 1200 cm^−1^ corresponded to C-N stretching and CH bending, whereas high levels in 1150–1000 cm^−1^ were associated with C-O and C-O-C vibrations attributed to carbohydrates and polysaccharides, with the 1150–1000 cm^−1^ peaks showing a significant increase in fermented groups (FMF and FHMF). The fingerprint region (900–500 cm^−1^) contained several sharp peaks that represented phosphate groups, mineral components, and structural changes in the matrices, with FHMF exhibiting the strongest spectral characteristics; thus, there was more chemical modification in it. Comprehensively, the comparative FTIR profiles indicate that the hydrolysate and fermentation (especially FNF) treatments enhanced the complexity of the molecules.

#### 3.1.5. Proximate Composition of Feeds and Fish

The nutritional profile of the hydrolysate-based and fermented feeds was generally similar to determined by proximate composition analysis. The crude protein content of both hydrolysate-based and fermented feeds was almost the same (49.41% and 49.63%, respectively), which implies that both feeds had a high protein content throughout. Otherwise, the levels of carbohydrates were approximately the same (32.67% in hydrolysate-based feed and 31.79% in fermented feed), while lipid content was 10.51% in hydrolysate-based feed and 10.97% in fermented feed. Both formulations had minimal amounts of ash (2.53% and 2.25%) and moisture (4.81% and 5.14%, respectively). A higher total energy content was obtained in the hydrolysate-based feed (3906.58 kcal/kg) than in the fermented feed (3806.58 kcal/kg) (Table 1).

The proximate composition of the fry was significantly different from the formulated feeds, mainly due to the high moisture content in the fish tissues. The hydrolysate-based feed had 25.41% protein, 3.24% carbohydrate, 3.17% lipid, 1.41% ash, and 66.97% moisture, while the fermented formulation had 25.22% protein, 2.58% carbohydrate, 3.29% lipid, 1.33% ash, and 68.19% moisture. The similarity of whole-body protein and lipid levels between the two growth groups suggests that nutrient deposition in the fry of the two groups is not different, though statistical analysis would be required to determine if the differences were due to the treatments (Table 1).

### 3.2. Growth Performance and Survival Rate

A substantial impact of Micro- and Nano-particulate Fermented Feed and Micro and Nano-particulate Hydrolysate based Feed have been observed on the *C. carpio* initial life stages’ growth potential, as depicted in Figure 5. A two-way ANOVA was used to analyze the impact of feed, age, and their interaction (feed × age) on four growth parameters such as weight gain percentage (WG%), final body weight (FB), net weight gain (NWG), and specific growth rate (SGR). Feed (F = 1008.9, *p* < 0.001) and Age (F = 9073.89, *p* < 0.001) had substantial effects on WG%, and there was a significant interaction effect (F = 419.00, *p* < 0.001, η^2^ = 0.01). The interaction term for FB was significant (F = 188.6, *p* < 0.001), with large effects from Feed (F = 3235.3, *p* < 0.001) and Age (F = 1989.722, *p* < 0.001). Feed (F = 4849.2, *p* < 0.001), Age (F = 2168.864, *p* < 0.001), and their interaction (F = 2570, *p* < 0.001, η^2^ = 0.01) all had significant effects on NWG. There was a statistically significant but relatively small interaction effect (F = 357.98, *p* < 0.001) between SGR and Feed (F = 460, *p* < 0.001) and Age (F = 1832.84, *p* < 0.001). Based on these data, both feed and age have a substantial impact on growth measures, and while interaction effects are statistically significant, their magnitude differs by parameter. The best performance of all growth parameters was observed in T-4, followed by T-3, while in the case of survival, both groups showed the highest survival percentage (Figure 6).

### 3.3. Intestinal Enzyme Activity

Figure 7 shows the effects of Micro- and Nano-particulate Fermented Feed and Micro- and Nano-particulate Hydrolysate-based Feed on the functioning of intestinal enzymes in *C. carpio* during the initial stages of life. Two-way ANOVA showed a positive influence of feed on protease activity (F = 40.9, *p* < 0.001), Lipase (F = 1278.88, *p* < 0.001), and cellulase activity (F = 16,695.28, *p* < 0.0001). Age also significantly affected intestinal enzyme activity and served as an additional independent variable (Protease activity, F = 49.87, *p* < 0.001; lipase, F = 345.56, *p* < 0.001; and Cellulase, F = 5,932,487, *p* < 0.0001). Furthermore, there was a very strong interaction (*p* < 0.0001) between feed and age on DAH. (Protease, F =, *p* < 0.001, Lipase, F =, *p* < 0.001 and Cellulase, F = 184.16, *p* < 0.0001) Explored the interaction and modulation of intestinal enzyme Activity by both independent factors. The data indicated that both factors (diet and age) and their interaction had a substantial and almost identical impact on protease, lipase, and cellulase activity. An investigation of primary impacts showed that the fermented nano feed (T-4) had the most significant impact on cellulase and lipase activity. However, the activity of protease varied. Each comparison between pairs of meals revealed a fluctuating pattern in the levels of intestinal enzymes DAH. T-4 > T-3 > T-2 > T-1 > control (Figure 7).

### 3.4. Histological Analysis

Figure 8 shows the effect of micro- and Nano-particulate Fermented Feed and micro- and Nano-particulate Hydrolysate-based Feed on the gut of common carp during early rearing. Moreover, all pairwise comparisons in experimental groups revealed that fermented nanofeed shows a higher effect on villi height, villi length, villi width, and absorptive area, followed by fish hydrolysate-based nanofeed, fermented microfeed, and hydrolysate-based microfeed, while the control shows the least effect.

### 3.5. Expression of Genes in C. carpio Supplemented with Different Prepared Diets

The two-way ANOVA results show significant differences in terms of gene expression, with various levels of interaction between them Figure 9. In the case of IGF, both feed (F = 706.2, *p* < 0.000) and age (F = 783.3, *p* < 0.000) caused large main effects, while the interaction between feed and age was relatively small and significant (F = 16.06, *p* < 0.000, η^2^ = 0.025). Feed (F = 1488.0, *p* < 0.000) and age (F = 263.1, *p* < 0.000) significantly affected GH expression, with a stronger interaction effect (F = 21.74, *p* < 0.000, η^2^ = 0.139). Ghrelin levels were significantly influenced by feed (F = 152.5, *p* < 0.000), age (F = 352.0, *p* < 0.000), and the feed-age interaction, which had the highest effect among all genes (F = 6.91, *p* < 0.000, η^2^ = 0.025). Feed (F = 467, *p* < 0.000) and age (F = 3.4, *p* = 0.006) influenced interleukin expression, with a minor but significant interaction (F = 1.63, *p* = 0.025, η^2^ = 0.029). Lysozyme had a substantial main effect of feed (F = 174.0, *p* < 0.000) and age (F = 309.0, *p* < 0.000), but no interaction (F = 1.46, *p* = 0.064, 2 η^2^ = 0.013). Feed h had a large impact on TNF expression (F = 225.3, *p* < 0.000), whereas age had a smaller influence (F = 7.49, *p* < 0.000). However, feed and age did not interact significantly (F = 1.25, *p* = 0.177, η^2^ = 0.010). These findings reveal that, while feed type continued to have a major influence on gene expression patterns, age also played a role, and the degree of feed-age interactions with specific genes varied, with the largest changes occurring with GH and ghrelin.

## 4. Discussion

The use of nanoparticulate feed technology has revolutionized aquaculture nutrition, especially in the early developmental stages when the nutritional needs of fish larvae are quite complex and cannot be met by conventional feed formulations. Larvae of common carp in the present 45-day feeding trial showed significant improvement in growth performance, digestive efficiency, and physiological development when fed micro- and nanoparticulate fermented and hydrolysate-based diets. The results indicate that high-quality particulate feeds could serve as a nutritious tool for commercial larviculture.

All the experimental feeds analyzed by scanning electron microscopy (SEM), X-ray diffraction (XRD), and Fourier-transform infrared spectroscopy (FTIR) to understand the characteristics responsible for these improvements. One of the biggest challenges of traditional feeds is that they are larger than the gap of small-mouthed fish larvae; this gap is the nanometer-size range required by the feed, which was achieved by SEM analysis, showing a consistent particle size distribution. One problem with the conventional larval feeds is that they have relatively large particle sizes, which may restrict ingestion by the small-mouth-opening fish larvae. The nano-particulate feed was developed to overcome this limitation, and SEM analysis showed a particle size range of 90–105 nm. The significant reduction in particle size can increase the digestibility of feed and make it easier for small-mouthed larvae to ingest, which is likely to increase feeding efficiency during the larval stage. This smaller particle size enhances the nutrient/water surface-area-to-volume ratio, which allows more interaction between nutrients and water, leading to faster nutrient dissolution. The structural characteristics are especially beneficial to *Cyprinus carpio* larvae as their digestive system is still developing, and they rely on readily ingested and efficiently digested feed particles [17].

The characteristics of the formulated feeds besides particle size could also have affected their nutritional performance. The result of the XRD analysis confirmed the crystalline nature of both fermented and hydrolysate formulations, which showed that the manufacturing process maintains the molecular integrity of the nanoparticles. The importance of crystal structure for maintaining the structure is that it helps to ensure a slow release of nutrients during digestion, so that there is a steady supply of the proper amino acids and fatty acids through the digestive process without significant nutrient leaching into the surrounding water. The controlled release can benefit utilization efficiency and decrease environmental losses of nutrients [28] (Figure 1).

The FTIR analysis also showed that the main functional groups of proteins, lipids, and carbohydrates remained unaffected by the preparation of the nanoparticles. The amide I and amide II bands around 1650 cm^−1^ and 1540 cm^−1^ were observed, indicating that the secondary structure of proteins was not significantly altered during the process of nanoparticle formation. The preservation of these structural features is crucial to retain protein quality and enzymatic accessibility during digestion [29]. In addition, the presence of the characteristic absorption bands also indicates successful incorporation of the bioactive compounds produced during the fermentation and hydrolysis process (Figure 4).

The feeding trial results demonstrated biological responses to the improved physicochemical properties of the experimental feeds. The results showed that all diets were superior to conventional feed, and improved growth-related parameters were obtained. Of these, the size of nanoparticles (around 90–105 nm) may have played a role in enabling ingestion (while minimizing prey capture and movement of food) and therefore had a positive effect on the feeding efficiency [30].

Another possible reason for the better growth response with the fermented diets is that bioactive compounds are produced during fermentation. Beneficial microorganisms produce metabolites (e.g., bioactive peptides and short-chain fatty acids) that have been linked to improved nutrient assimilation and growth hormone release. Similarly, protein hydrolysates have been shown to induce expression of amino acid transporters, such as LAT2 and PepT2, and stimulate the TOR signaling pathway, which can increase protein synthesis and somatic growth [31].

One of the most important factors in intensive aquaculture is larval nutrition, and the nutritional and physiological requirements of fish larvae change rapidly during their early development, which means that the diets must meet these needs. However, there is still limited knowledge of the nutritional requirements of larvae, the development of the digestive system throughout ontogeny, the formulation of efficient feed, particle size, and feed texture, which still limits the development of efficient feeds for larvae [32]. Thus, nutritionally balanced and highly palatable feeds are necessary to ensure good larval growth and survival in the hatchery.

Larval diets have various physical properties that can influence feed acceptance and use, especially particle size. Due to their small mouth gape, fish larvae are unable to ingest large feed particles efficiently. The experimental diets in the present study were thus ground into micro- and nanoparticulate size fractions to enhance the uptake and assimilation of nutrients. In addition to particle size, the nutritional demand of larvae also varies during ontogeny with the development of organ systems [33]. In fish, the use of carbohydrates as an energy source is limited, especially glucose [34]. In the same way, dietary lipids are essential to normal larval growth and development, and other combined nutritional and environmental factors influence successful larval rearing [10]. Therefore, it is important to formulate species-specific and age-specific larval diets for maximum survival and healthy development.

Another mechanism by which early nutrition interventions impact physiological performance is via nutritional programming. Appropriate dietary stimulation at critical life stages causes metabolic changes that continue through the rest of life [35,36]. The same approach has been used before in predatory marine fish to enhance their ability to use dietary carbohydrates [37]. Balanced formulated diets have also been successfully developed for rohu larvae more recently. The present study compared the use of different feed formulations (fermentation and hydrolysate) as nutritional programming methods and their effects on feed utilization by larval fish. In addition, because nutrient leaching is a frequent problem with nano-sized feeds, this process was added to the feed production to enhance nutrient stability and retention in the feed (Figure 6).

The diets were then prepared and their structural features analyzed by Scanning Electron Microscopy (SEM) and X-ray Diffraction (XRD). SEM showed that the nanoparticulate feeds had a particle size between 90 and 105 nm and a smooth surface with uniform characteristics. This particle size is found to be very appropriate for feeding the larvae, as the smaller the particle size, the easier for them to ingest, the more available nutrients are to them, and the better they will be absorbed by their digestive system. In addition, the uniform surface morphology seen in the SEM images shows good uniformity of particles and structural stability, which are desirable traits for larval feed formulations. In addition, XRD analysis was performed to verify that the prepared nanoparticles had a crystalline structure, which further supports the structural integrity and potential functional stability of the nanoparticles.

The particle size produced by the present study is in good agreement with previous studies. Yablon [38] reported nanoencapsulated nutrients for freshwater aquaculture ranging from 50 to 100 nm, and Akhtar [39] showed that nanoparticles with a size of 70–90 nm significantly enhanced nutrient accessibility and larval growth performance. Similarly, Mourdikoudis [40] pointed out that particle size and shape are crucial factors in the performance of nanoparticles, while Shivalkar [41] noted that XRD plays a crucial role in determining the crystalline structure of nanocomposite materials. All these studies corroborate the findings of structural features observed in the present study and validate the suitability of the developed particulate feeds for larval cultivation.

The nutrient concentration of the experimental feeds also supported this suitability as nutrient-rich larval food. The EDX analysis showed a high percentage of carbon and oxygen in both fermented feed (FF) and hydrolysate-based feed (HF), suggesting that the carbon and oxygen are mainly organic substances, which are a good source of energy and nutrients for developing larvae. In fact, FF was found to be 49.1% carbon and 27.8% oxygen, while HF was found to be 45.7% carbon and 30.1% oxygen. These were the dominant components, but both feeds were also rich in nitrogen, calcium, potassium, sodium, chlorine, and selenium, which are nutritionally important minerals. The elemental composition of FF consisted of nitrogen (21.6%), calcium (0.7%), potassium (0.4%), sodium (0.3%), chlorine (0.4%), and selenium (0.3%), while HF contained nitrogen (22.6%), calcium (0.5%), potassium (0.4%), sodium (0.3%), chlorine (0.2%), and selenium (0.2%).

All of these are physiologically important for supporting growth, antioxidant protection, bone mineralization, enzyme activation, metabolic regulation, and osmoregulation. The elemental profile results reported in the present study agree with previous reports on nutritionally balanced larval feeds and their influence on water quality. Furthermore, biofortification by fermentation has been reported to increase the bioavailability of amino acids and decrease the presence of antinutritional factors [16], and the supplementation of bioactive peptides in hydrolyse-based diets has been found to improve digestibility, nutrient utilization, and immune responses [18]. All these results indicate the nutritional adequacy of both experimental diets and their use in freshwater larval-rearing aquaculture.

The formulated feeds were further analyzed based on their molecular properties using Fourier-transform infrared spectroscopy (FTIR). FTIR spectra showed the presence of typical absorption bands for the functional groups related to the proteins, lipids, and other organic compounds, which indicated that the major biochemical constituents had not been affected after the feed processing. The characteristic carbonyl, alkyl, and Si–O stretching vibrations are like those reported in the characterization of polymer nanocomposites and nanomaterials [41]. The close similarity between the present results and those of previous studies suggests that the structure of the prepared particulate feeds was well preserved during the feed formulation process. After the structure determination, it is important to include the nutritional importance of hydrolysis and fermentation. It is generally accepted that both processing methods enhance the nutritional value of aquafeeds by making more protein digestible and bioavailable, and bioactive peptides. For the present study, enzymatic hydrolysis was chosen as a method to develop a functional larval diet because developing fish larvae have a strong need for a balanced amino acid profile and readily digestible peptides for their quick growth rate.

Enzymatic hydrolysis has several nutritional advantages over chemical hydrolysis. Acid hydrolysis can also give rise to high levels of sodium chloride and deterioration of nutritive elements, like the destruction of a certain amino acid (tryptophan), which can lower the nutritional value of fish protein hydrolysates (FPH) [42]. Alkaline hydrolysis does not destroy tryptophan but may destroy other amino acids like serine and threonine. However, enzymatic hydrolysis is more likely to reduce the degradation of amino acids, and the enzymatic hydrolysates do not contain unwanted racemization products. The fish protein hydrolysates (FPH) have also been reported to have less than 5% fat content [43] and have better storage stability compared to the hydrolysates with high fat content [44]. Efficient hydrolysis also yields a wide variety of amino acids, and enzymatic hydrolysis is superior in preserving the nutritional integrity as compared to chemical hydrolysis [45]. In addition, the resulting papain- and Proteinase K-derived hydrolysates have lower quantities of fat and moisture, making them desirable ingredients for large-scale production of feed [46].

In summary, these results show that hydrolysate- and fermented-based feeds contain ingredients with nutritional value to support larval development and growth. Some slight differences were found between the two formulations, but the fermented feed always had slightly superior nutritional properties to the hydrolysate diet. This is in line with the observations made in previous studies and confirms that fermentation is an effective method to be used successfully to enhance larval feed quality (Figure 7).

Biological advantages of experimental diets were manifested in the growth performance of common carp larvae. The fermented nanoparticulate feed (FNF) was able to achieve the maximum improvements in growth and survival among all the treatments, which is beneficial due to the synergy between the two processes. The growth enhancement effects of fermented diets have been observed in aquaculture species earlier [16]. Similarly, Soengas [47] showed that the type of feed and the age of the larvae had significant effects on growth performance and emphasized the potential role of nanotechnology in boosting feed use efficiency and growth efficiency in aquaculture.

Larvae fed the experimental diets showed digestive enzyme activities significantly improved, which suggests that more efficient use of nutrients by larvae fed the experimental diets. Both micro- and nanoparticulate formulations activated the digestive capacity, with the highest responses always being seen with the FNF treatment. The highest protease activity was observed in FNF, which indicates better digestion of protein and higher availability of amino acids. Lipase activity was also found to be the highest in T-4, showing increased digestibility of lipids, which are important for energy storage, membrane formation, and normal growth of larvae.

The present results confirm several earlier studies [48] that found that the fermented mahua oil cake significantly enhanced growth, nutrient utilization efficiency, and digestive enzyme activities in *L. rohita*. Similarly, yellow-feather chickens (YFCs) fed fermented diets showed an improvement in digestive enzyme activity, intestinal morphology, and immune response [49]. Previous work has also shown direct effects of dietary protein level and protein source on digestive enzyme activity in fish larvae and digestive enzyme stimulation by specific dietary additives in early life [50]. Furthermore, nutrient-specific diets are important for digestive development, while enzyme activities are known to vary naturally with larval development. All these studies point strongly to the improvement in digestive function reported here.

Digestive enzyme activity is an accepted measure of efficient digestion and utilization of nutrients in fish feed [51]. Previous studies showed that fermented diets resulted in a significantly higher activity of proteases, particularly a 75% fermented tuna offal meal, which had the highest activity (0.62 IU mL^−1^) [52]. Microbial activity during fermentation is responsible for much of the enhanced digestive capacity of fermented feeds, compared to non-fermented feeds. The beneficial microorganisms Bacillus spp. can degrade complex proteins, carbohydrates, and lipids into simpler compounds such as amino acids, peptides, and fatty acids through the actions of proteases, lipases, and chitinases. Protease activity further enhances protein availability by breaking down complex proteins into free amino acids and short peptides, and microbial biomass provides additional protein by assimilation of inorganic nitrogen [53]. These smaller molecules are easily absorbed through the intestinal epithelium and quickly enter circulation, where they could promote the synthesis of protein, tissue development, and biomass production. In addition, during fermentation, the proteins become more available for fish due to their peptides and amino acids that prevent the growth of microorganisms in the process of spoilage.

Protein hydrolysates have also been proven to have beneficial effects on digestive physiology. The effect of substituting 25% of fish meal with fish-muscle protein hydrolysate on the pancreatic enzymes (trypsin, chymotrypsin, and lipase) was demonstrated in *Carassius auratus gibelio* by [54]. Likewise, Hlordzi [55] found that diets that included 37% hydrolyzed fish protein (HFP) significantly increased trypsin and chymotrypsin activity in pearl gentian grouper due to the low-molecular-weight peptides it contained, which stimulated the secretion of digestive enzymes. Kwasek [29] Higher plasma amino acid levels and greater protease activity after feeding amino acid-rich diets to channel catfish. Overall, the results of the present study are confirmed by the recent findings that hydrolysate-based diets containing a high content of di- and tripeptides rapidly increase digestive enzyme secretion in both gastric and agastric fish.

The experimental diets had positive effects on the development of digestive enzymes, growth performance, and intestinal development of common carp larvae. Intestinal maturation in the early rearing period was enhanced in fish fed with nanoparticulate diets, as evidenced by the significant increase in villus length, villus width, tunica muscularis thickness, and absorptive surface area. The structural modification proposed in this study indicates that nanoencapsulation technology can be beneficial to the health of the gut by enhancing the intestinal surface area for absorbing nutrients and thus improving the digestive efficiency and growth of larvae.

The current results confirm the beneficial effect of the use of advanced feed formulations on intestinal morphology, as illustrated in previous works. Co-fermented diets containing probiotics significantly enhanced gut development and nutrient assimilation in the largemouth bass (LMB) [56]. Likewise, Misra [57] demonstrated an increase in nutrient digestibility in cultured fish due to fermentation. The probiotic diets in the form of nano-encapsulated probiotics also reported the improvement of the length and width of the villi, which can result in better intestinal health and absorption of nutrients [58]. Moreover, De Marco[59] showed that improved feed formulations had more advantages in the development of the gut than traditional feed. All these studies support the changes in intestinal architecture found in the present investigation and underscore the importance of feed structure on efficient nutrient utilization during the larval stage (Figure 1 and Table 2).

In the present study, therefore, the potential mechanisms behind the growth performance were further investigated by analyzing the expression of important growth-related genes in the common carp larvae. Among the endocrine pathways that control somatic growth, the growth hormone–insulin-like growth factor (GH–IGF) axis is a major player in teleosts, where growth hormone (GH) stimulates insulin-like growth factor-1 (IGF-1) to promote growth [60]. Larvae in treatment T4 had the most positive GH, IGF-1, and ghrelin expression levels, indicating that this dietary treatment might have been responsible for increasing essential nutrient availability and utilization and increasing endocrine signaling related to growth regulation. The coordinated increase in these growth-related genes further supports the notion that the formulated diet was better for initiating the GH–IGF axis, thus aiding in the higher performance of the T4-treated larvae (Figure 9).

The upregulation of these genes is in line with the previous reports on physiological advantages of nano-formulated feeds. Here, it has been demonstrated that nano-sized foods can influence the expression of genes related to growth and metabolism by enhancing nutrient delivery and bioavailability. Orexin, a feed intake, energy balance, and growth-controlling orexigenic hormone, is especially sensitive to improvements in nutritional status [61]. Similarly, de Moraes [62] showed that by using nano-formulated diets, appetite-related hormones can be regulated to allow increased feed intake and growth performance. The significant upregulation of IGF-1 expression level in T-4 cells also supports the enhanced growth response seen in the present study, which is consistent with earlier studies that have shown that expression of IGF-1 is significantly enhanced by nano-encapsulation technology and enhances aquaculture productivity [17].

In addition to growth control, experimental diets also had a significant effect on immune-related gene expression. The treatment with T-4 always resulted in the highest expression of lysozyme, interleukin, TNF, TGF-β, IL-1, and IL-1β, which showed an increase in innate immune competence of all treatments. Nano-formulated diets with improved nutrient stability and controlled release could enhance the availability of biologically active compounds that can boost immune function and thus improve the initial immune response of fish larvae.

The results are in line with earlier studies, which showed immunostimulatory effects of functional and nano-formulated aquafeed. Rajendran [63,64] have reported that Nano-formulated fermented feeds enhance the bioavailability of bioactive compounds that are responsible for enhancing immune responses. Likewise, Lu [65] showed that immune-related gene expression in fishes can be affected by the type and method of feed. The developmental variation in the present study is also consistent with [66], which found that developmentally regulated variation in immune gene expression occurs naturally in aquatic animals. Furthermore, Mokhtar [67] highlighted the importance of immune cells and cytokines in fish immunity, and Zhang [54] showed that immune-associated genes were significantly expressed in fish larvae fed with functional diets supplemented with bioactive ingredients. These results indicate that there is sufficient support for the improvement in immune response seen in common carp larvae fed experimental diets.

This study has several limitations to consider when interpreting the results. First, particle characterization was limited to size range and morphology by SEM; polydispersity index, zeta potential, and dispersion stability/nutrient leaching over time were not measured, so the physical stability of the nano- and micro-particulate feeds in water over the feeding period is not fully established. Finally, the associations drawn between the XRD/FTIR structural characterization and functional outcomes (digestive enzyme activity, growth) are correlative rather than mechanistically demonstrated, and the trial was conducted at laboratory scale over 45 days, so extrapolation to commercial hatchery conditions or later developmental stages requires further validation; moreover, the feed intake efficiency should be further studied to estimate the exact value.

## 5. Conclusions

Among the four experimental diets, the fermented nanoparticulate feed (FNF) produced the highest growth, survival, digestive enzyme activity, villus development, and expression of growth- and immune-related genes in *C*. *carpio* larvae under the conditions tested. Reducing particle size to the nanoscale, combined with fermentation, was associated with greater apparent nutrient utilization than the microparticulate or hydrolysate-based formulations in this trial. These findings indicate that fermented nanoparticulate feed is a promising candidate larval diet for common carp, though the improvements observed here are specific to the 45-day, laboratory-scale conditions of this study, and given the limitations outlined above, should be confirmed under longer-term, larger-scale, and commercial hatchery conditions before broader practical or production recommendations can be made. Future work should prioritize characterizing particle stability and hydrolysate composition directly, monitoring water quality more completely at high stocking densities, and evaluating gut microbiota and nutrient metabolism across a longer rearing period.

## Figures and Tables

**Figure 1:**
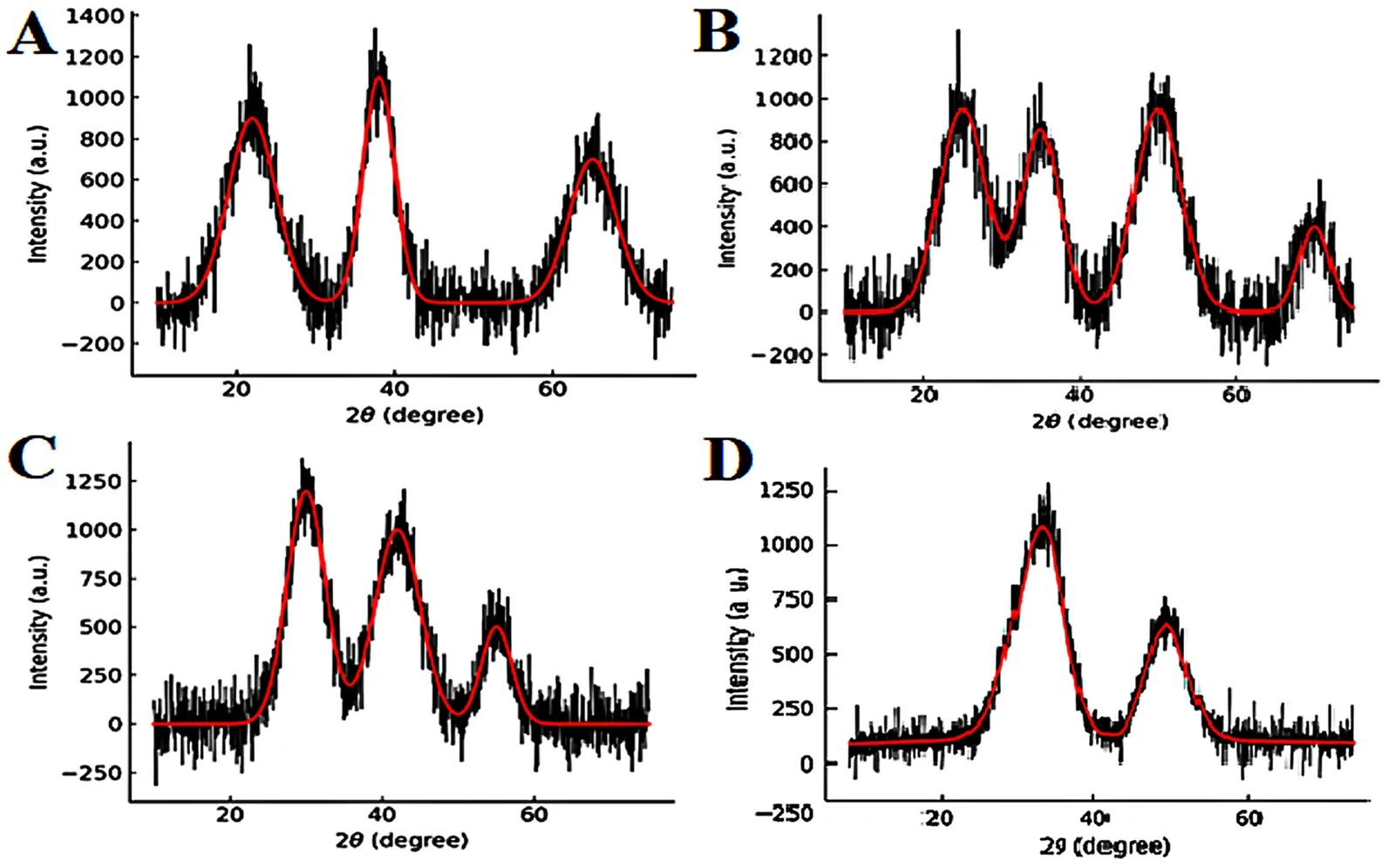
X-ray diffraction (XRD) patterns of the four experimental diets: (**A**) fish hydrolysate-based micro feed (FHMF), (**B**) fermented micro feed (FMF), (**C**) fish hydrolysate-based nano feed (FHNF), and (**D**) fermented nano feed (FNF), show variations in diffraction peak characteristics among the different feed formulations.

**Figure 2:**
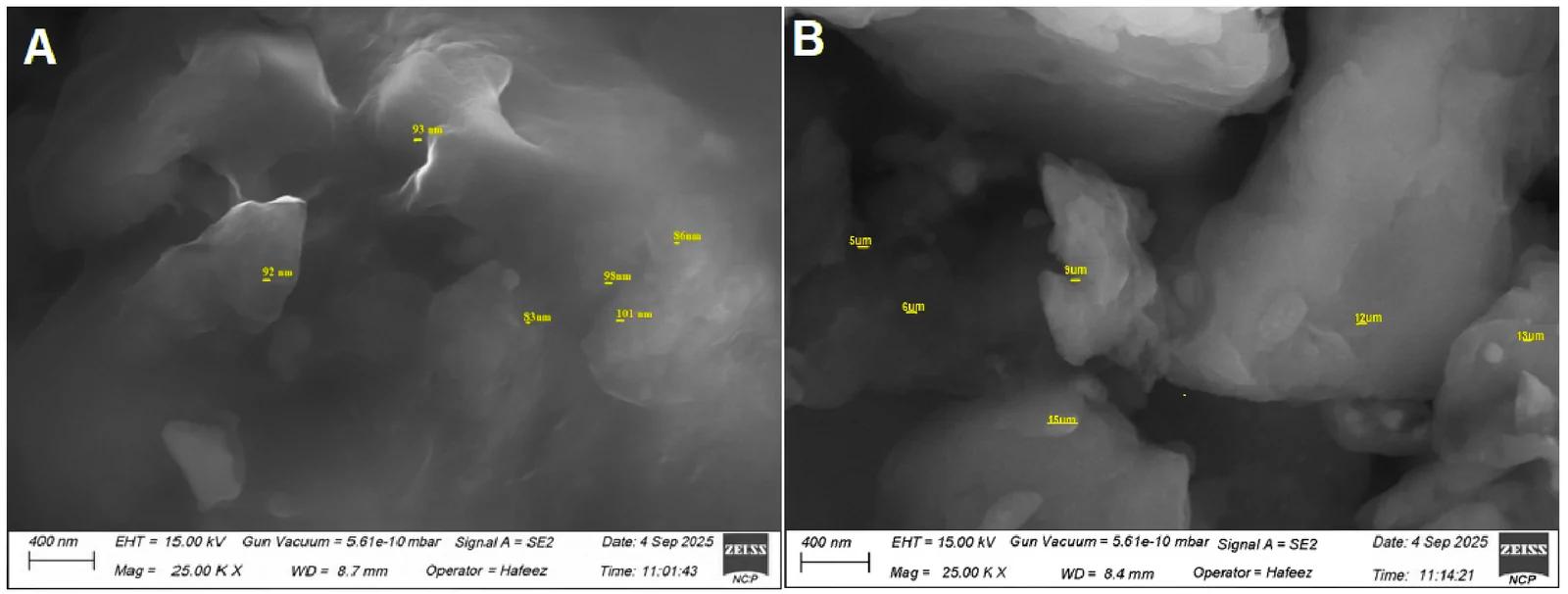
SEM analysis of (**A**) Nano-particulate larval feed and (**B**) Micro-particulate larval feed.

**Figure 3:**
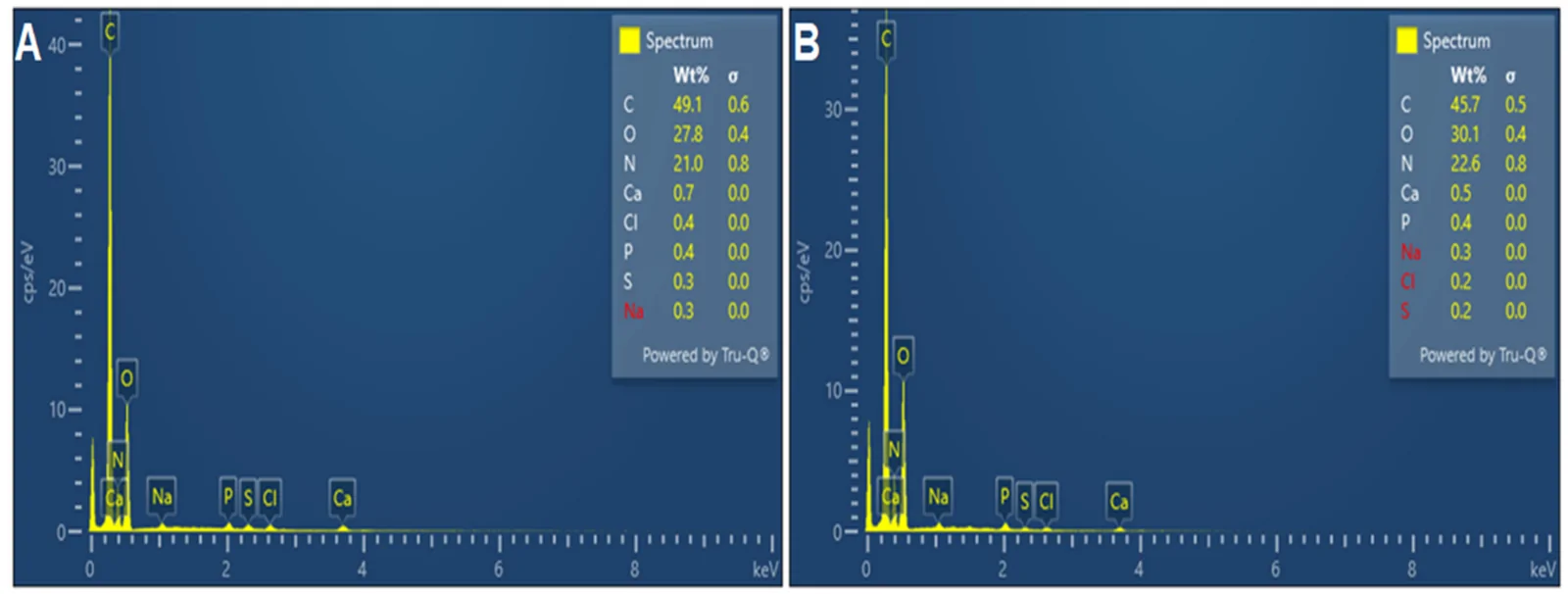
EDX analysis of different prepared diets for the *C. carpio* larvae: (**A**) Fermented larval feed and (**B**) Hydrolysate-based larval feed.

**Figure 4:**
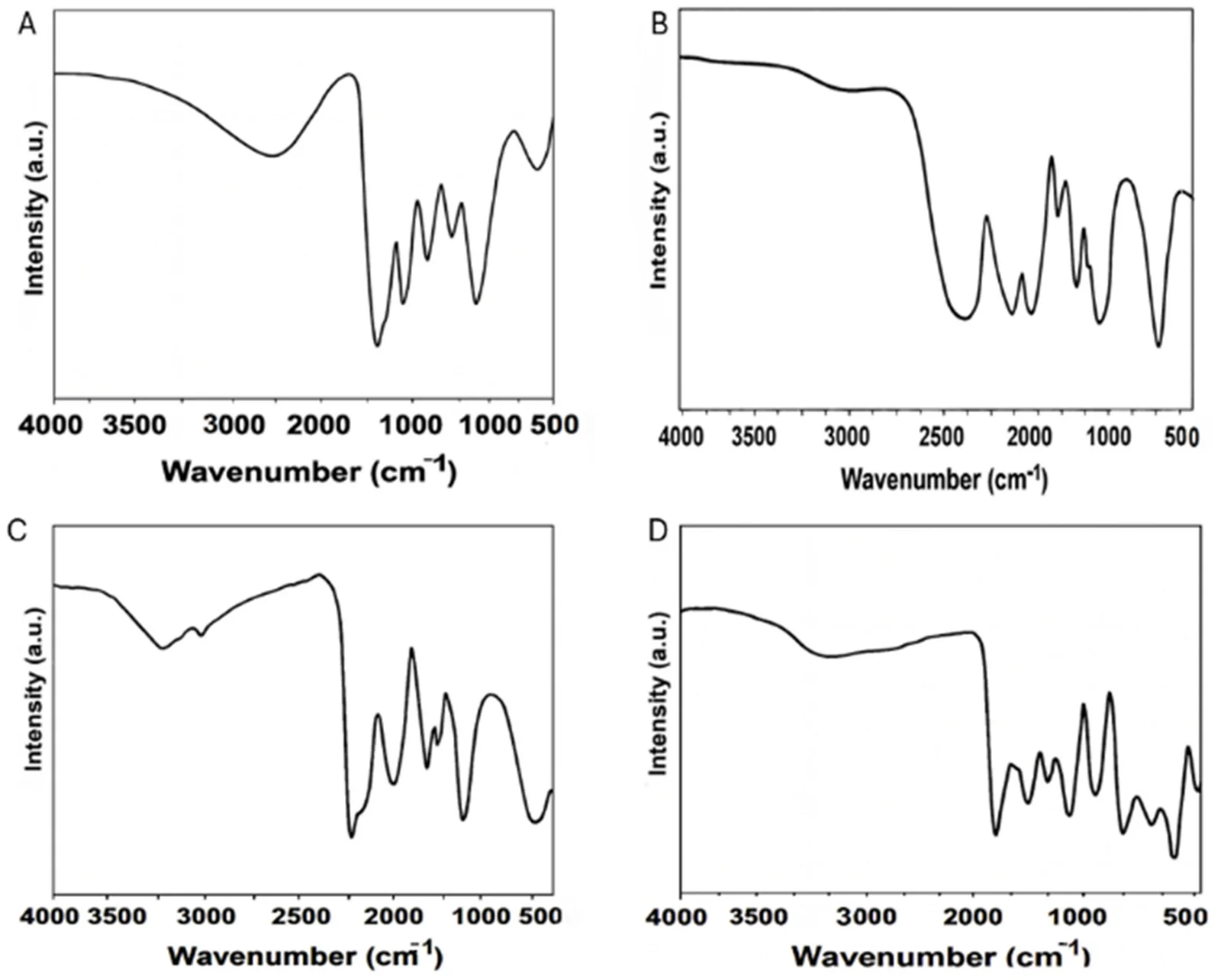
FTIR pattern of different prepared diets: (**A**) FNF, (**B**) FHNF, (**C**) FMF, and (**D**) FHMF.

**Figure 5:**
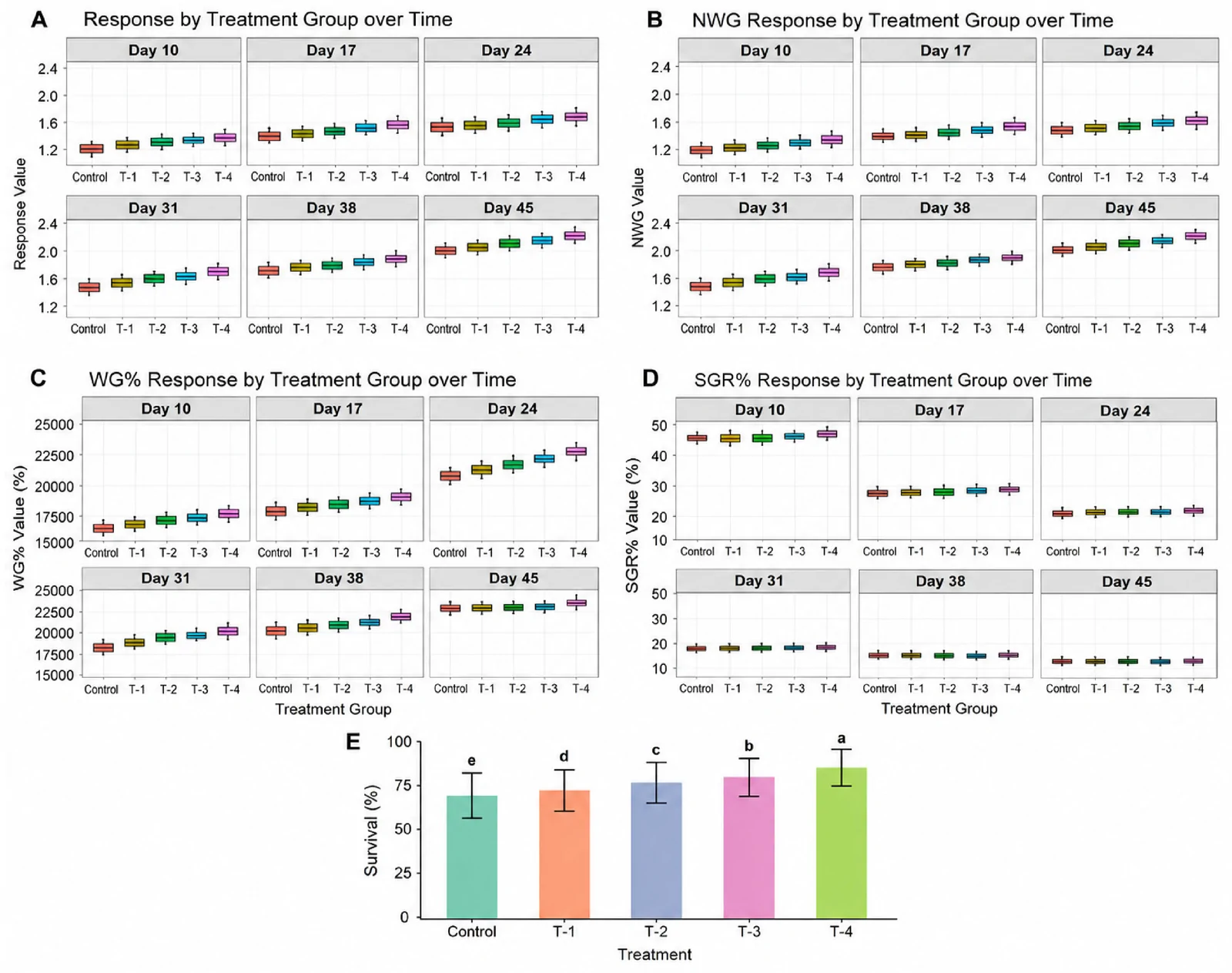
(**A**) Final Body Weight, (**B**) Net Weight Gain, (**C**) Weight Gain Percentage, and (**D**) Specific Growth Rate Percentage. Data were analyzed using two-way ANOVA with Feed type and Age as independent variables, and post hoc pairwise comparisons were performed to assess inter- and intra-group differences (*n* = 9). Values are presented as means (PLs weight values in grams). (**E**) Survival percentage of *C. carpio* PLs after a 45-day feeding trial.

**Figure 6:**
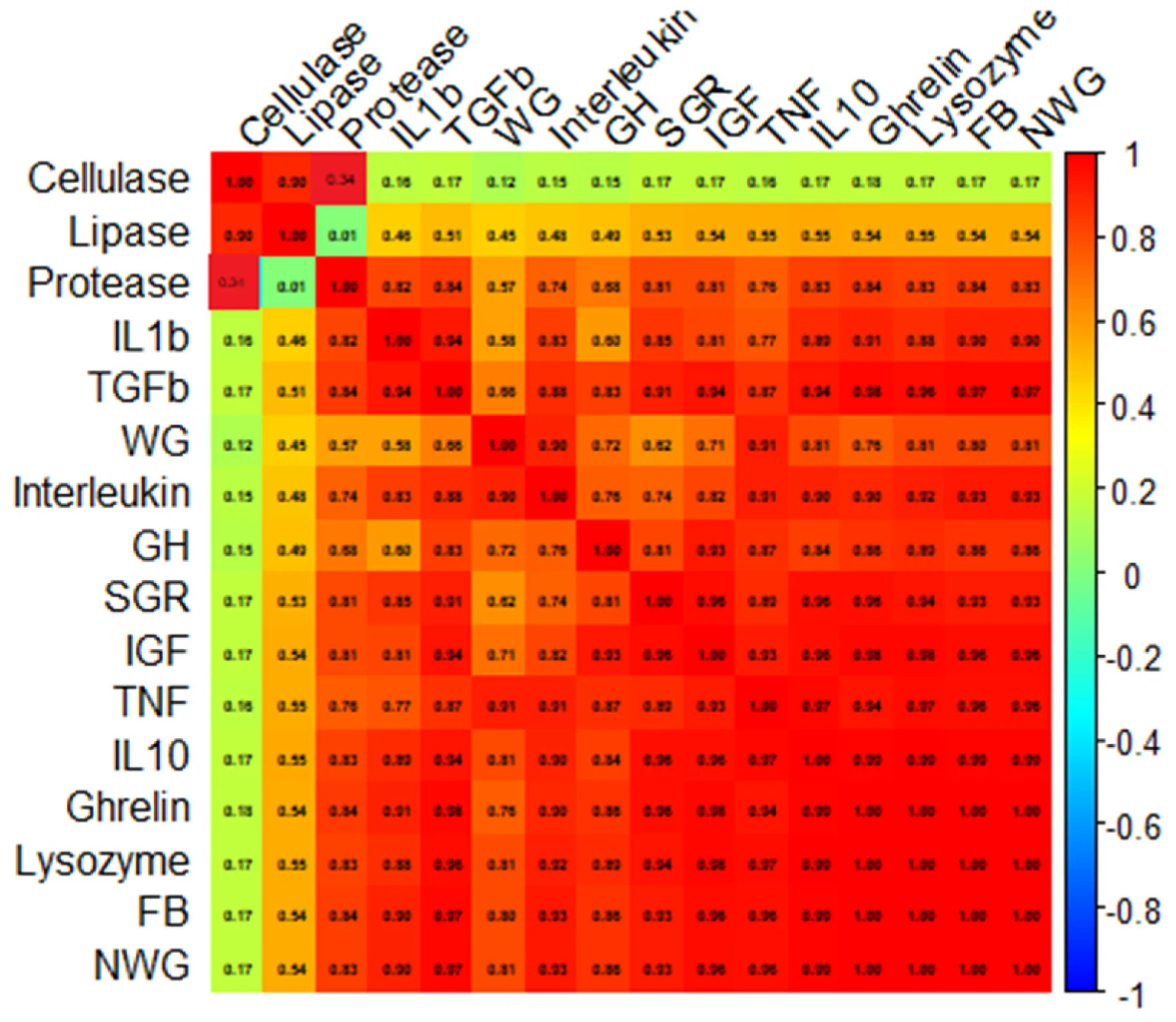
Pearson correlation analysis integrating all measured variables (Net weight gain, Weight Gain, Final Body weight, SGR%, Survival%, Protease, Cellulase, Lipase, Ghrelin, IGF-1, GH, Interleukins, IL10, IL1beta, TGF beta, Lysozyme) at 5 different treatments during the exposure period. The numbers indicate the Pearson correlation coefficient. All the indices showed a positive correlation. A number close to 1 represents a higher positive correlation.

**Figure 7:**
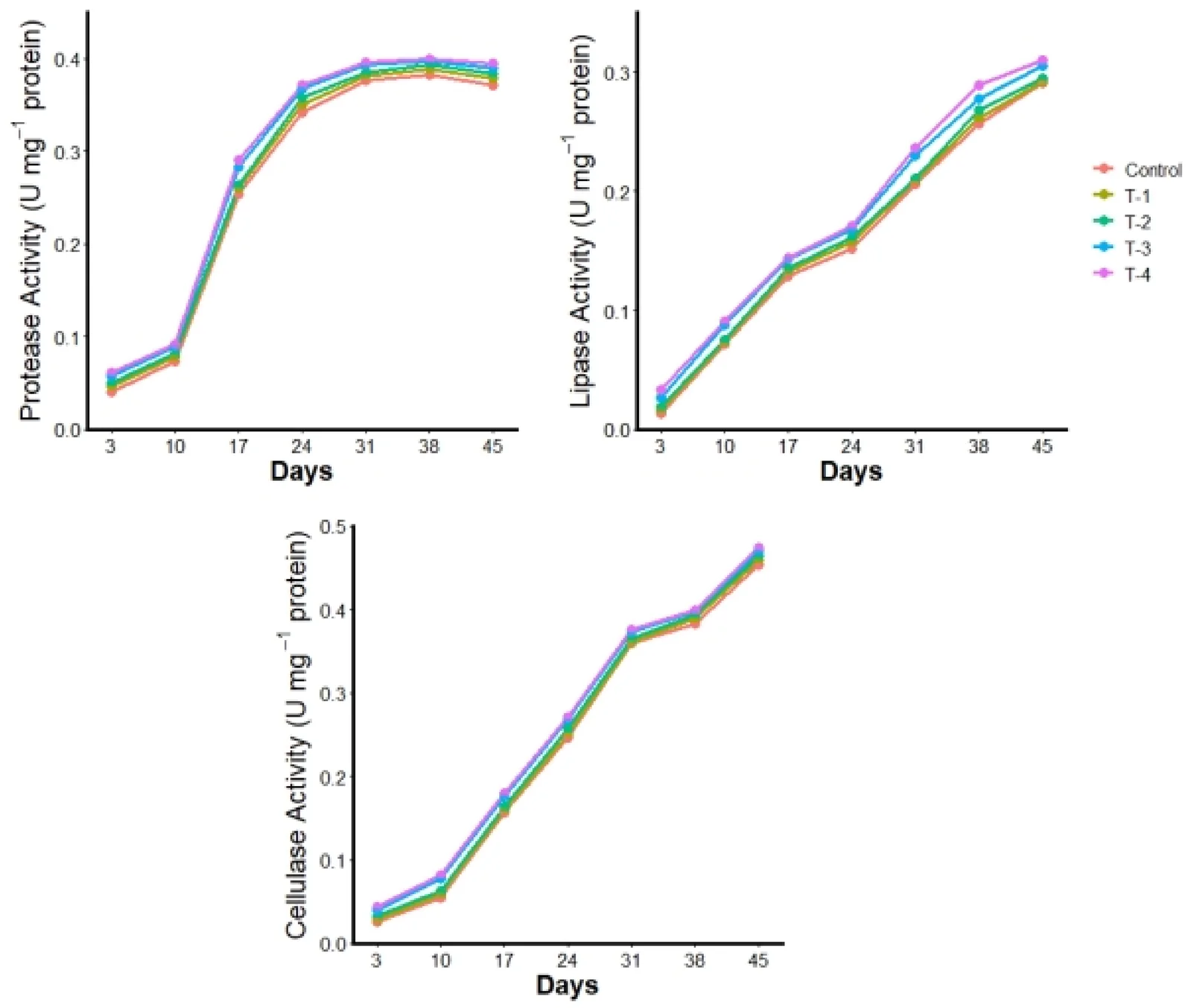
Data on digestive enzymes (Protease, Cellulase, and Lipase) were analyzed using two-way ANOVA with Feed Type and Age (DAH) as independent variables, followed by post hoc pairwise comparisons to identify significant differences both between and within treatment groups (*n* = 9). Values are presented as means (U mg^−1^) ± SEM. Different subscript letters within the same row denote statistically significant differences (*p* < 0.05) among feed types (inter-group comparisons) at each age. Superscript letters within the same column indicate age-related differences in enzyme activity within each feed type (intra-group comparisons).

**Figure 8:**
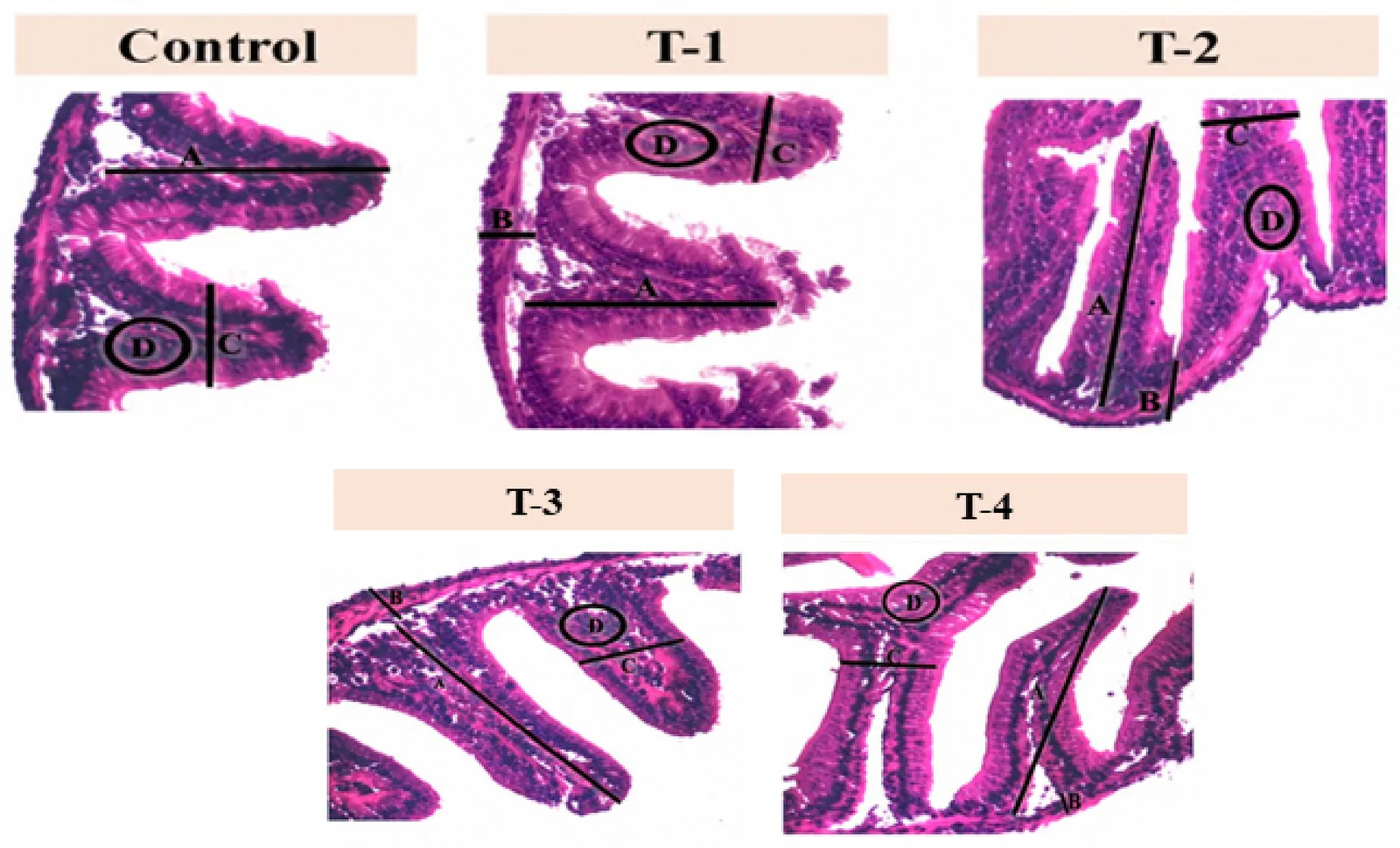
Microscopic analysis of the intestine of *C. carpio* larvae after feeding on experimental diets for 35 days. A = villi length; B = width; C = tunica muscular thickness; D = Absorptive area.

**Figure 9:**
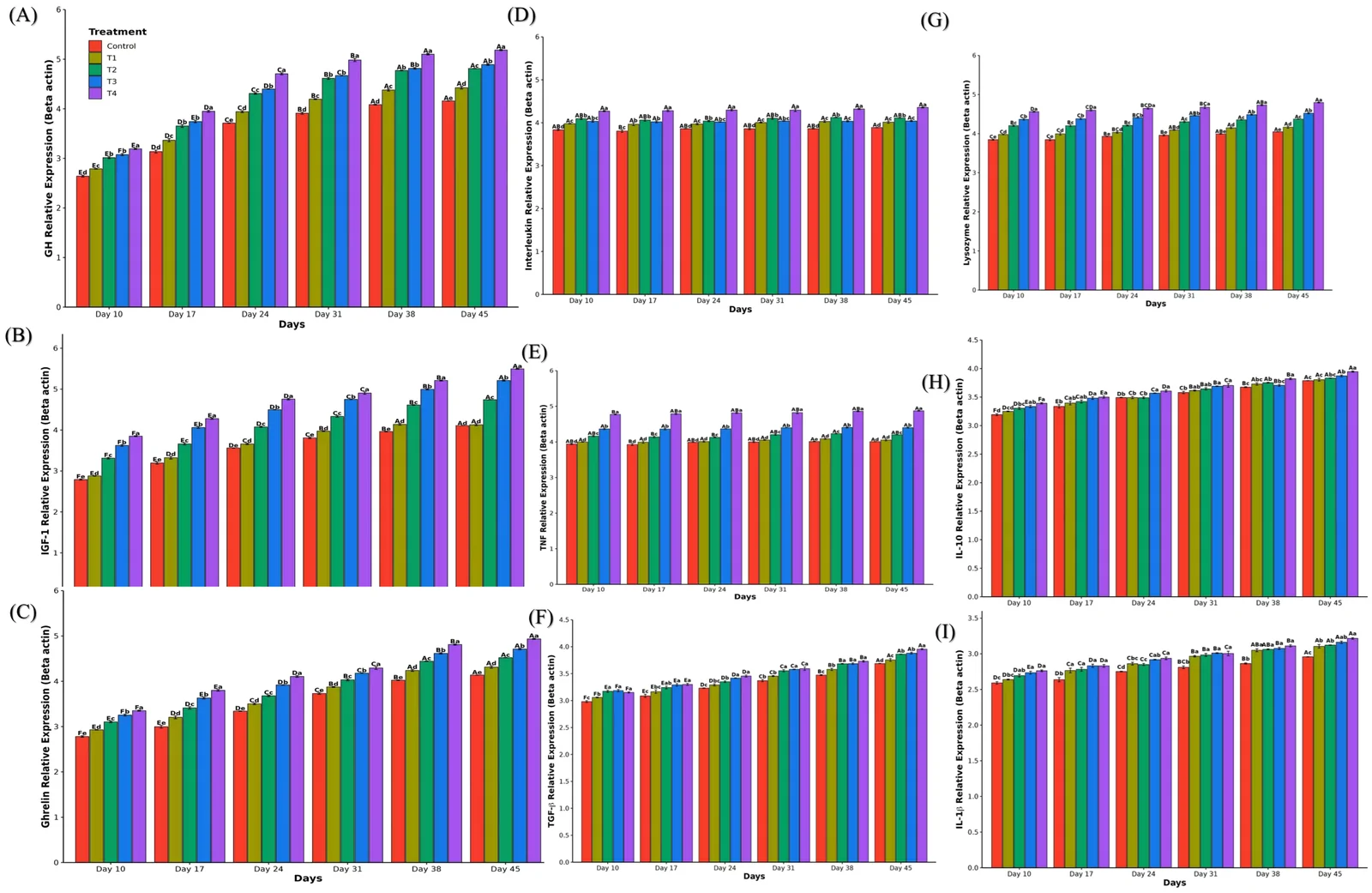
Expression of (**A**) GH, (**B**) IGF-1, (**C**) Ghrelin, (**D**) interleukin IL-1β, (**E**) TNF-α, (**F**) TNF-β, (**G**) lysozyme, (**H**) IL-10, and (**I**) IL-1β, with β-Actin as the reference gene. Results are expressed as mean relative expression (normalized to β-Actin) ± SE. A two-way ANOVA followed by LSD post hoc tests was used to assess the effects of feed type and age (Days After Hatching, DAH) on their expression. Different lowercase superscripts indicate significant differences among feed types at a given age (*p* < 0.05), while uppercase letters denote significant differences across ages within the same treatment.

**Table 1:** Proximate composition (%) of fish hydrolysate-based feed, fermented feed, and fries reared on the prepared diets.

Groups	Crude Protein (%)	Carbohydrates (%)	Lipid (%)	Ash (%)	Moisture (%)	Total Energy (kcal/kg)
Hydrolysate-based Feed	49.41	32.67	10.51	2.53	4.81	3906.58
Fermented Feed	49.63	31.79	10.97	2.25	5.14	3806.58
Fry (HF)	25.41	3.24	3.17	1.41	66.97	
Frys (FF)	25.22	2.58	3.29	1.33	68.19	

**Table 2:** Shows the changes in villous morphology during the 45-day experiment.

Parameters	Control	T-1	T-2	T-3	T-4	F-Value	*p*-Value	SEM
Villi length (µm)	520 ^d^	691 ^c^	705 ^bc^	721 ^b^	757 ^a^	328091.4	0.000	69.7
Villi width (µm)	351 ^d^	425 ^c^	404 ^c^	455 ^b^	495 ^a^	68299.7	0.000	29.43
Tunica Muscularis (µm)	141 ^d^	288 ^c^	301 ^b^	311 ^b^	334 ^a^	28669.5	0.000	19.06
Absorptive area (µm^2^)	477 ^d^	558 ^c^	551 ^c^	658 ^b^	701 ^a^	41170.2	0.000	36.12

Values are presented as mean ± SEM. The alphabets show significant differences. Data were analyzed using two-way ANOVA (*n* = 9).

## Data Availability

The original contributions presented in this study are included in the article/Supplementary Materials. Further inquiries can be directed to the corresponding author.

## Supplementary Materials

The following supporting information can be downloaded at: https://www.mdpi.com/article/10.3390/biology15191680/s1, Table S1. Composition of fish hydrolysate-based larval feed. Table S2. Composition of fermented fish feed. Table S3. Primers used to evaluate the mRNA expression of the studied genes. Table S4. Summary of XRD characteristics of experimental diets.
