# Supplementary material for: Effect of Nano- and Microparticulate Fermented Fish Protein Hydrolysate Diets on Growth, Digestive Function, Intestinal Development, and Gene Expression in Common Carp (Cyprinus carpio) Larvae
Source: Biology (Basel). 2026 Sep 22;15(19):1680. doi: 10.3390/biology15191680 (PMC13663553; doi:10.3390/biology15191680)
Supplement: Supplementary file 1 [file biology-15-01680-s001.zip › biology-4486845-supplementary.pdf]

Table S1. Composition of fish hydrolysate-based larval feed.

Ingredients	CP%	Inclusion level (g/100g)	CP % level	DE (kcal/kg diet)
Fish hydrolysate	60	50	30	2,225.00
Soybean meal	46	42	19.32	1,354.08
Rice polish	10	2	0.3	112.50
Wheat flour	14	1.5	0.28	35.00
Vitamin and mineral premix		2	0	0
CMC		0.5	0	0
Fish oil		2	0	180.00
Total		100	49.73	3,906.58

Table S2. Composition of fermented fish feed.

Ingredients	CP%	Inclusion (g/100g)	CP level (%)	DE (Kcal/kg)
Fish meal	60	50	30	2,125.00
Soybean meal	46	42	19.32	1,354.08
Rice polish	10	2	0.2	112.50
Wheat flour	14	1.5	0.28	35.00
Vitamin and mineral premix		2	0	0
CMC		0.5	0	0
Fish oil		2	0	180.00
Total		100	49.73	3,806.58

Table S3. Primers used to evaluate the mRNA expression of the studied genes.

Gene	Forward	TM	Accession number
**Beta actin**	F-GGCATGGGACAGAAGGACT	59.7 °C	XM_019089433.2
	R-AACACGCAGCTCGTTGTAGA	59.7 °C	
**Ghrelin**	F-TTTGCTTGTGTGTCGAGTCTG	58.8 °C	XM_019071738.2
	R-TGGAGCACTCACCATAAACTG	58.8 °C	
**IGF 1**	F-CGCCTCGAGATGTATTGTGCAC	60.5 °C	AF465830.1
	R-CTGTATGCCGTTGCGCTCGT	60.5 °C	
**GH**	F-TAGTGCTGTTGTCGGTGGTG	60.2 °C	AJ640135.1
	R-GGCAACAGGCTATCCTCAAA	60.2 °C	
**Interleukin**	F-CTCTCTTCCTGTCTGCCGTA	59.9 °C	XM_019073058.2
	R-GAACCTCGTCCCCAGATGT	59.9 °C	
**TNF-α**	F-AGGAGGATTGCTGCCCTTAC	59.7 °C	AJ311800.2
	R-TGCCTTGGAAGTGACATTTG	59.7 °C	
**Lysozyme**	F-GTGTCTGATGTGGCTGTGCT	59.7 °C	XM_019104788.2
	R-GTTCTTTCCACCTGGAGTGC	59.7 °C	
**TGF-β**	F-ACGCTTTATTCCCAACCAAA	60.0 °C	AF136947.1
	RGAAATCCTTGCTCTGCCTCA	59.8°C	
**IL-10**	F-CTCCGTTCTGCATACAGAGAAA	59.5 °C	JX524550.1
	R-TCATGACGTGACAGCCATAAG	59.6 °C	
**IL-1β**	F-TTACAGTAAGACCAGCCTGA	60.1 °C	XM_019080073.2
	R-AGGCTCGTCACTTAGTTTGT	59.9 °C	

All primers are arranged in the 5 ′ to 3 ′ direction.

Table S4. Summary of XRD characteristics of experimental diets.

Treatment	No. of major peaks	Approximate peak positions (2θ, °)	Dominant peak
FHMF	3	~22, ~38, ~64	~38°
FMF	4	~22, ~32, ~49, ~69	~49°
FHNF	3	~29, ~41, ~53	~29°
FNF	2	~33, ~47	~33°
